# Supplementary material for: Deterministic Formation of Single Organic Color Centers in Single-Walled Carbon Nanotubes
Source: Nano Lett. 2025 Aug 21;25(35):13103–9. doi: 10.1021/acs.nanolett.5c02378 (PMC12412172; doi:10.1021/acs.nanolett.5c02378)
Supplement: Supplementary file 1 [file nl5c02378_si_001.pdf]

Supporting Information for "Deterministic Formation of Single Organic Color Centers in Single-Walled Carbon Nanotubes"

Daichi Kozawa,[*,†,‡,¶] Yuto Shiota,[‡,§] Mengyue Wang,[†,‡] and Yuichiro K. Kato[*,†,‡]

†*Quantum Optoelectronics Research Team, RIKEN Center for Advanced Photonics, Wako, Saitama 351-0198, Japan*

‡*Nanoscale Quantum Photonics Laboratory, RIKEN Pioneering Research Institute, Wako, Saitama 351-0198, Japan*

¶*Research Center for Materials Nanoarchitectonics, National Institute for Materials Science, Tsukuba, Ibaraki 305-0044, Japan*

§*Department of Applied Physics and Physico-Informatics, Keio University, Yokohama, Kanagawa 223-8522, Japan*

E-mail: kozawa.daichi@nims.go.jp; yuichiro.kato@riken.jp

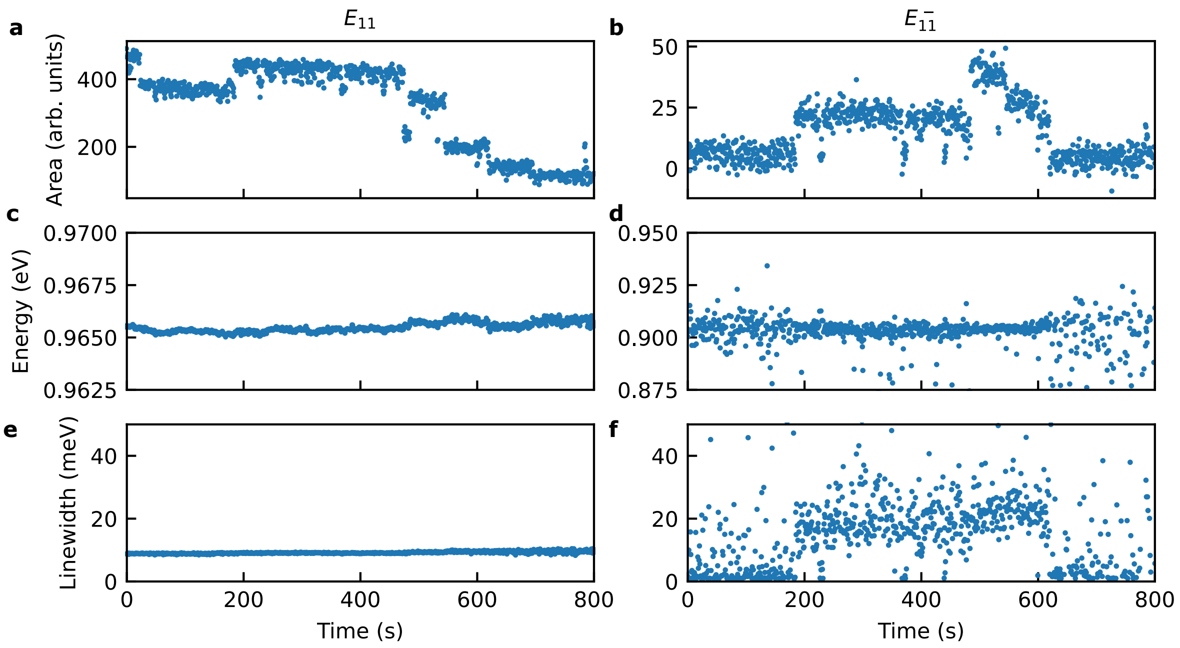

Figure S1: Time traces of (a, b) the peak area, (c, d) the emission energy, and (e, f) the linewidth of E_{11} and E_{11}^{-} peaks obtained by fitting bi-Lorentzian function to the spectra shown in Fig. 1b. The peak parameters are determined to be emission energies of 0.9650 $\pm$ 0.0002 eV and 0.902 $\pm$ 0.065 eV for E_{11} and E_{11}^{-} , respectively along with linewidths of 9.23 $\pm$ 0.26 meV and 26.9 $\pm$ 19.7 meV.

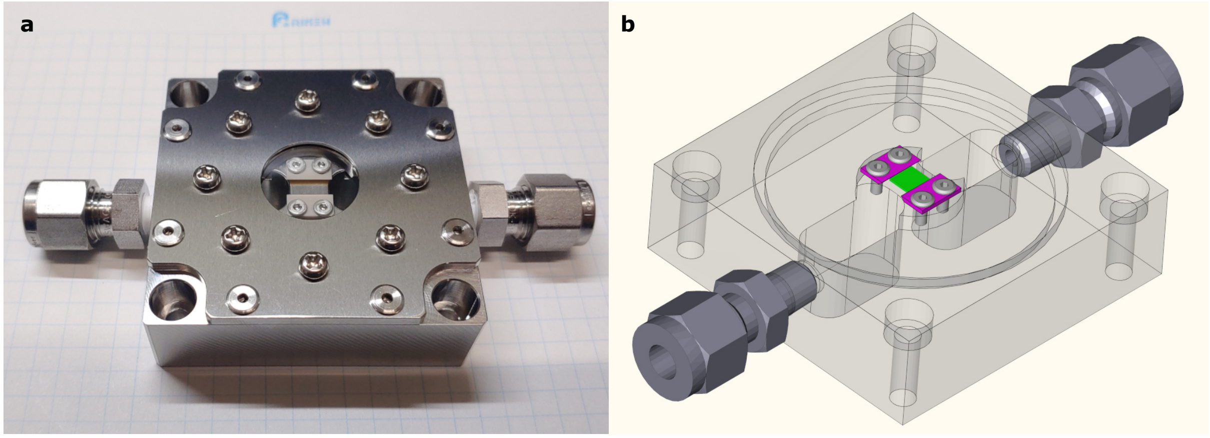

Figure S2: A picture and technical drawing of the gas-reaction cell where the tube fittings are closed during the reaction.

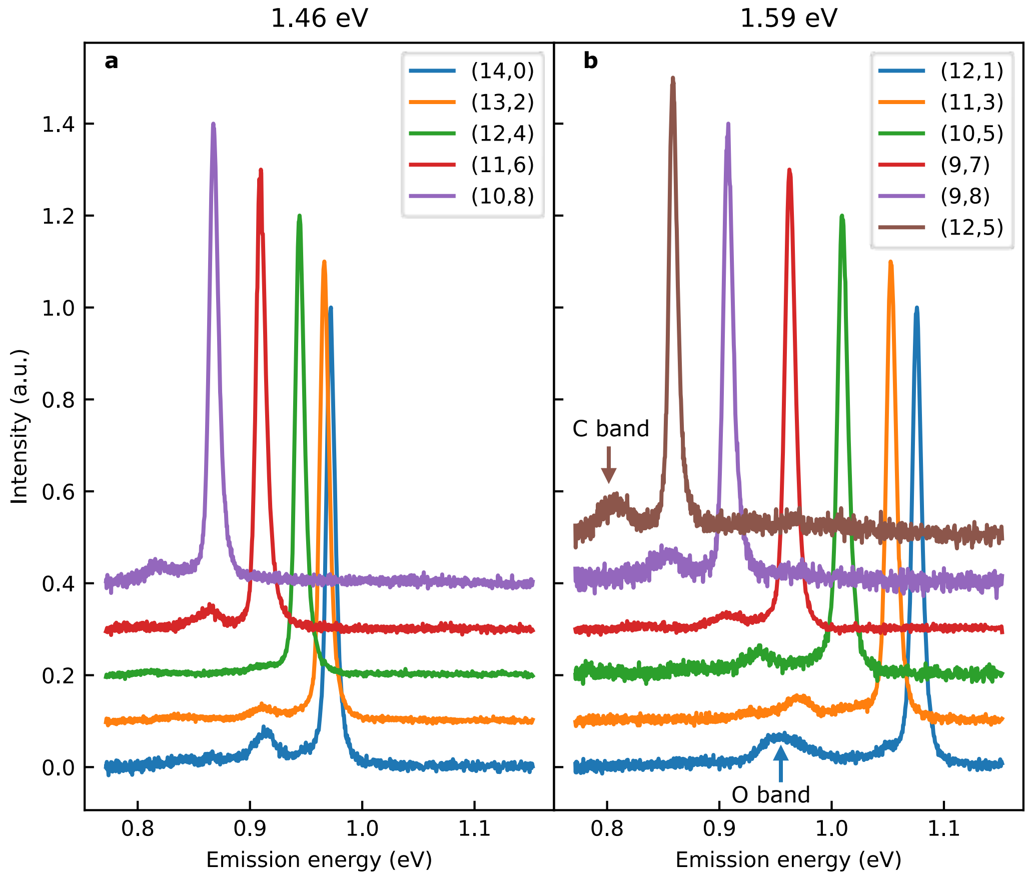

Figure S3: PL spectra of functionalized SWCNTs with chiralities of (14,0), (13,2), (12,4), (11,6), (10,8), (12,1), (11,3), (10,5), (9,7), (9,8), and (12,5). The spectra are collected with excitation laser energies of (a) 1.46 and (b) 1.59 eV and with an excitation power of 20 µW, where the spectra are displaced vertically for clarity. A color center formed in (12,1) nanotube emits in the O band corresponding to 1300 nm, and a color center formed in (9,7) nanotube emits in the C band corresponding to 1550 nm as indicated by arrows.

1 Reaction scheme

The photochemical reaction of iodobenzene with single-walled carbon nanotubes (SWNTs) proceeds as follows (Fig. S4):

Step 1 - Photoexcitation: Iodobenzene absorbs UV light with an energy of 4.09 eV, reaching an excited singlet or triplet state: Ph-I + hν $\rightarrow$ Ph-I*.

Step 2 - Homolytic bond cleavage: In the excited state, the relatively weak C–I bond in iodobenzene undergoes homolytic bond cleavage, generating radicals: Ph-I* $\rightarrow$ Ph$^\bullet$ + I$^\bullet$.

Step 3 - Radical addition: The phenyl radical subsequently forms a covalent bond with the nanotube sidewall through radical addition to the sp^2 carbon framework, creating the organic color center: SWNT + Ph$^\bullet$ $\rightarrow$ SWNT-Ph.

Iodobenzene is obtained from Fujifilm Wako Pure Chemical Corporation with greater than 97% purity and is used without further purification.

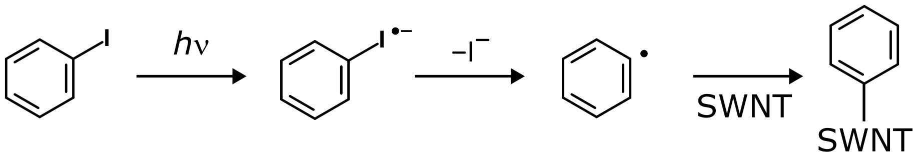

Figure S4: A reaction scheme of the photochemical reaction.

2 Control experiments of the functionalization

The potential formation of oxygen-related defects is examined, which provides insight into generated color centers. We conduct control experiments in air without iodobenzene on 9 nanotubes over 1200 s, and no color center emission has been observed Fig. S5. This provides direct evidence that iodobenzene is essential for the formation of organic color centers.

Additionally, we perform control experiments under dry N_2 instead of air in a closed cell to minimize oxygen and water vapor. Seven different (9,7) nanotubes are functionalized with iodobenzene, obtaining a formation rate of color centers of 0.014±0.018 s^{-1} (Fig. S6), which is consistent with the functionalization in air. The emission energies of E_{11}^{-} and E_{11}^{-*}

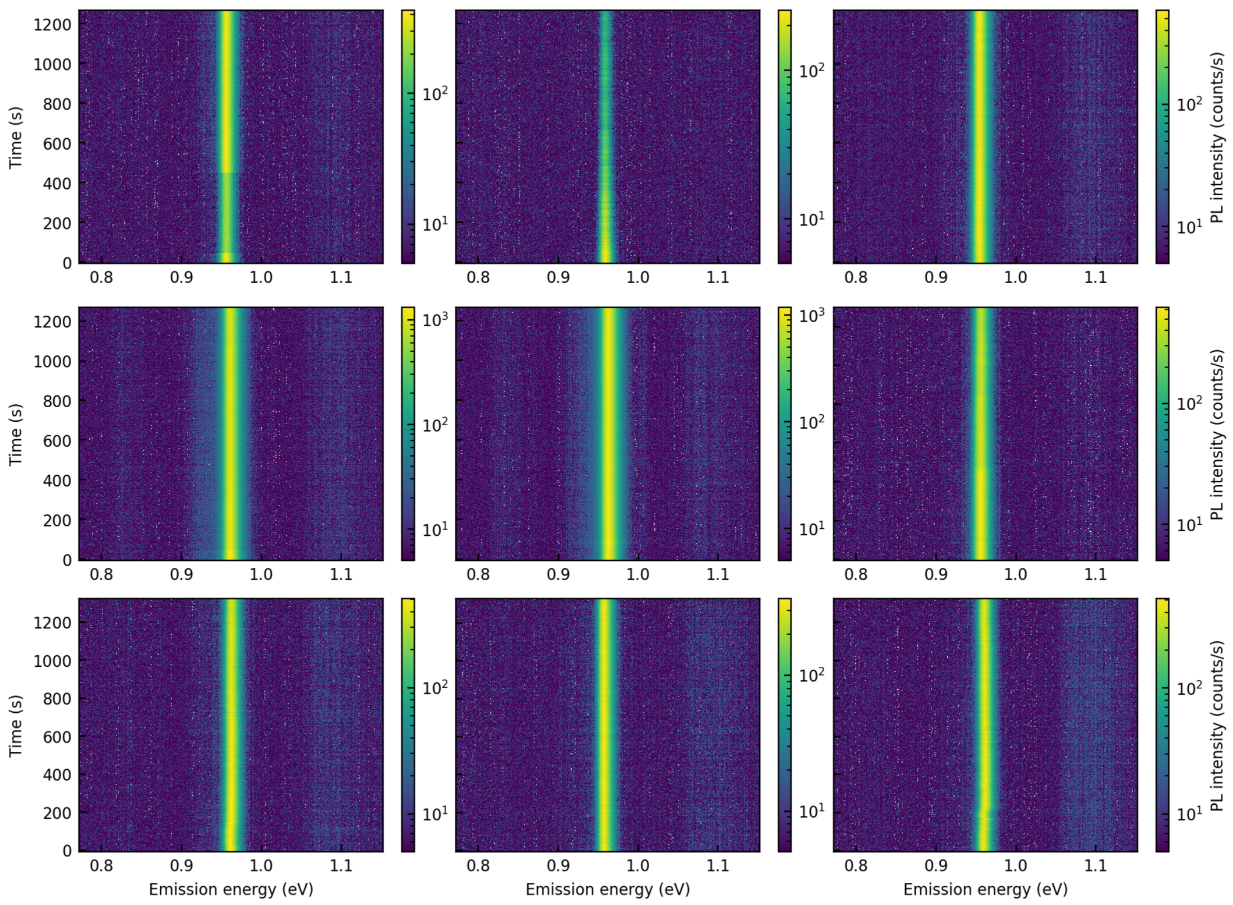

Figure S5: Time-trace maps of PL spectra for (9,7) SWNTs excited with 1.59 eV and 100 µW. The functionalization is conducted with a UV laser power of 5 nW without iodobenzene.

under these conditions are 0.867±0.007 eV and 0.839±0.006 eV, respectively, which are also consistent with the functionalization results in air (Fig. 3b). These results confirm that oxygen and water do not play a major role in the reaction.

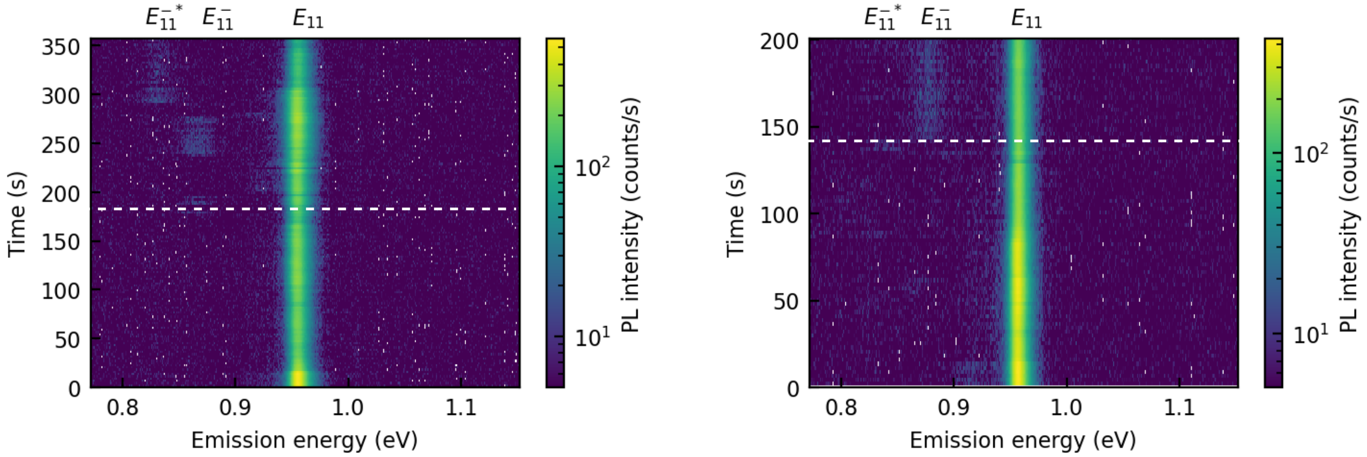

Figure S6: Representative time-trace maps of PL spectra for two separate (9,7) SWNTs excited with 1.59 eV and 100 µW. The functionalization is conducted with a UV laser power of 5 nW under dry N_2 environment. The first formation of a color center is indicated by broken horizontal lines.
